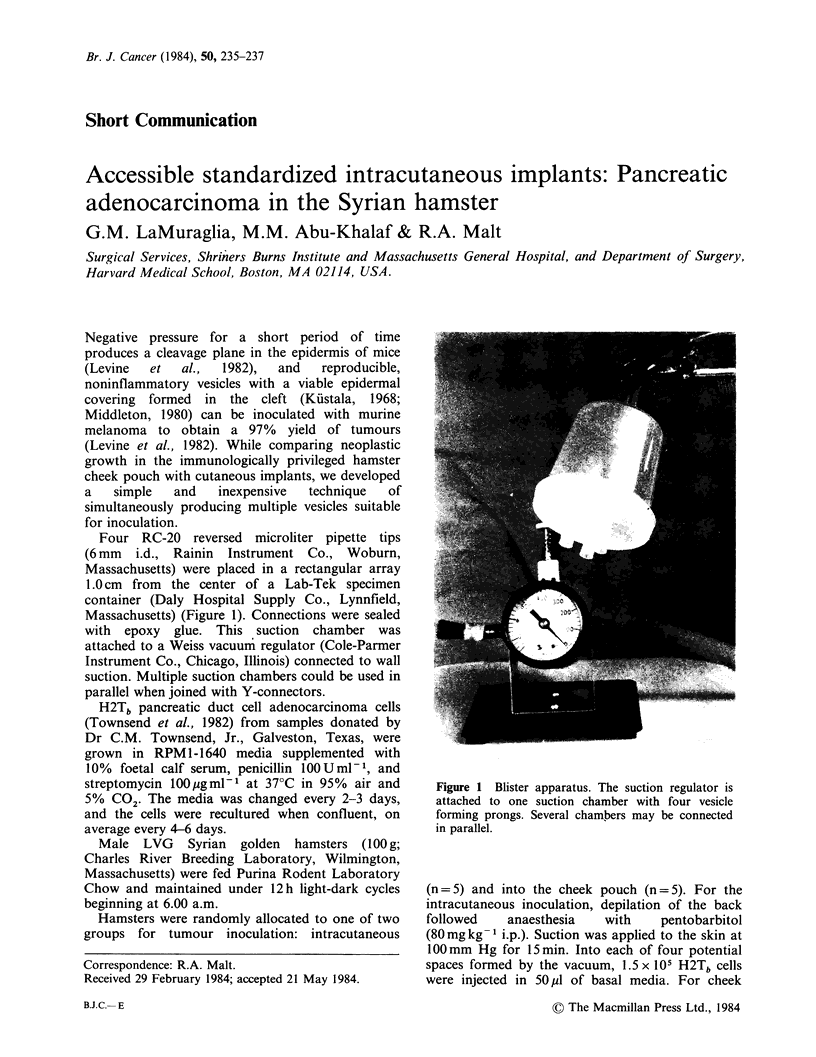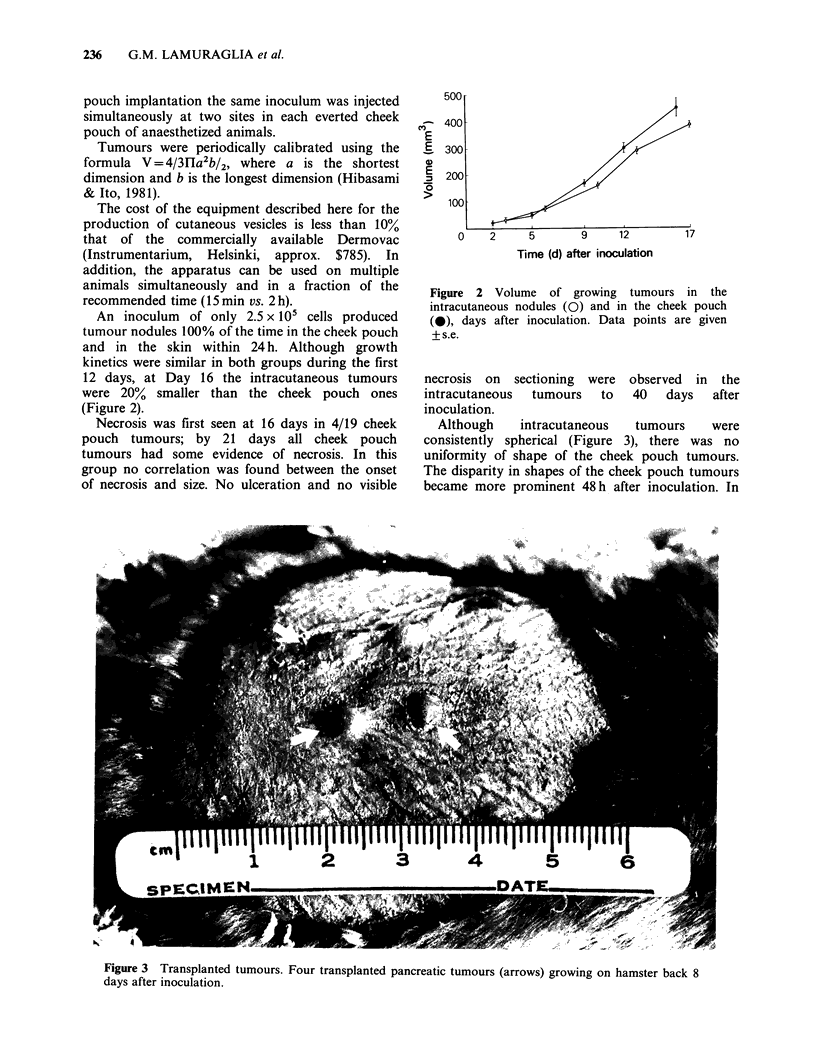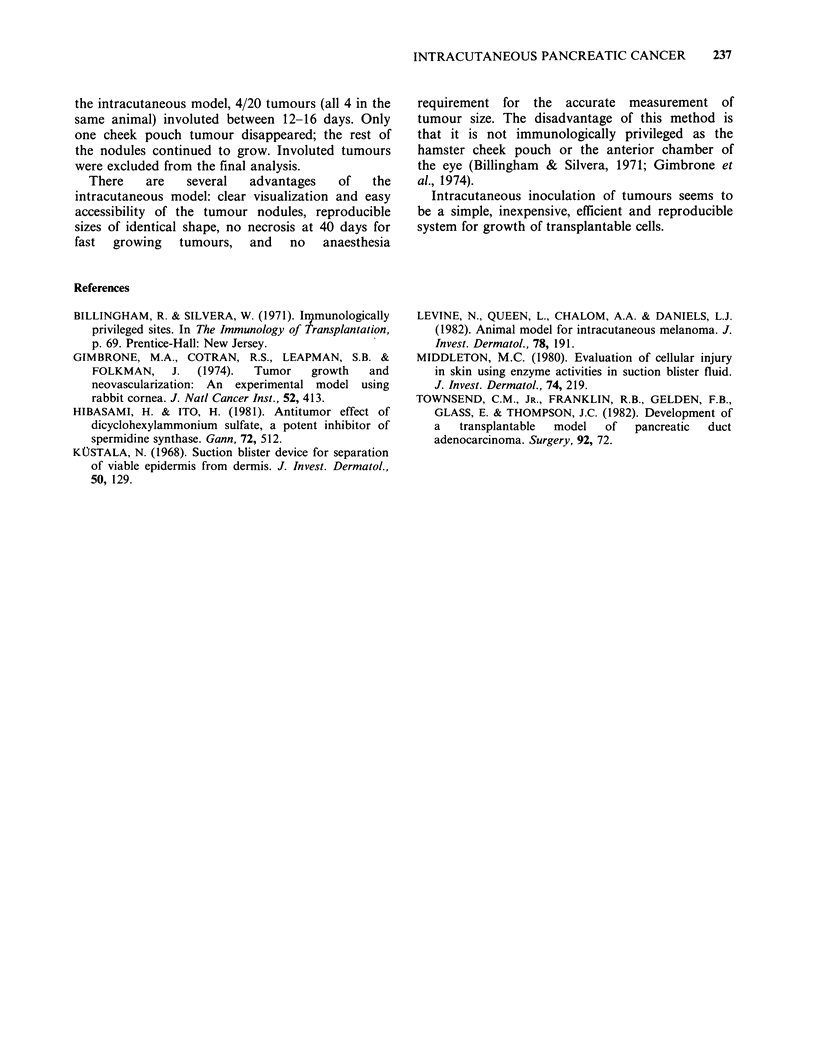# Accessible standardized intracutaneous implants: pancreatic adenocarcinoma in the Syrian hamster.

**DOI:** 10.1038/bjc.1984.169

**Published:** 1984-08

**Authors:** G. M. LaMuraglia, M. M. Abu-Khalaf, R. A. Malt

## Abstract

**Images:**


*Br. J. Cancer* (1984), **50**, 235–237

### Short Communication

### Accessible standardized intracutaneous implants: Pancreatic adenocarcinoma in the Syrian hamster

G.M. LaMuraglia, M.M. Abu-Khalaf & R.A. Malt

*Surgical Services, Shriners Burns Institute and Massachusetts General Hospital, and Department of Surgery, Harvard Medical School, Boston, MA 02114, USA.*

Negative pressure for a short period of time produces a cleavage plane in the epidermis of mice (Levine *et al.*, 1982), and reproducible, noninflammatory vesicles with a viable epidermal covering formed in the cleft (Küstala, 1968; Middleton, 1980) can be inoculated with murine melanoma to obtain a 97% yield of tumours (Levine *et al.*, 1982). While comparing neoplastic growth in the immunologically privileged hamster cheek pouch with cutaneous implants, we developed a simple and inexpensive technique of simultaneously producing multiple vesicles suitable for inoculation.

Four RC-20 reversed microliter pipette tips (6 mm i.d., Rainin Instrument Co., Woburn, Massachusetts) were placed in a rectangular array 1.0 cm from the center of a Lab-Tek specimen container (Daly Hospital Supply Co., Lynnfield, Massachusetts) (Figure 1). Connections were sealed with epoxy glue. This suction chamber was attached to a Weiss vacuum regulator (Cole-Parmer Instrument Co., Chicago, Illinois) connected to wall suction. Multiple suction chambers could be used in parallel when joined with Y-connectors.

$H2T_b$ pancreatic duct cell adenocarcinoma cells (Townsend *et al.*, 1982) from samples donated by Dr C.M. Townsend, Jr., Galveston, Texas, were grown in RPMI-1640 media supplemented with 10% foetal calf serum, penicillin $100\,U\,ml^{-1}$, and streptomycin $100\,\mu g\,ml^{-1}$ at 37°C in 95% air and 5% $CO_2$. The media was changed every 2–3 days, and the cells were recultured when confluent, on average every 4–6 days.

Male LVG Syrian golden hamsters (100 g; Charles River Breeding Laboratory, Wilmington, Massachusetts) were fed Purina Rodent Laboratory Chow and maintained under 12 h light-dark cycles beginning at 6.00 a.m.

Hamsters were randomly allocated to one of two groups for tumour inoculation: intracutaneous (n = 5) and into the cheek pouch (n = 5). For the intracutaneous inoculation, depilation of the back followed anaesthesia with pentobarbitol ($80\,mg\,kg^{-1}$ i.p.). Suction was applied to the skin at 100 mm Hg for 15 min. Into each of four potential spaces formed by the vacuum, $1.5\times10^5$ $H2T_b$ cells were injected in $50\,\mu l$ of basal media. For cheek

**Figure 1** Blister apparatus. The suction regulator is attached to one suction chamber with four vesicle forming prongs. Several chambers may be connected in parallel.

Correspondence: R.A. Malt.
Received 29 February 1984; accepted 21 May 1984.

© The Macmillan Press Ltd., 1984

pouch implantation the same inoculum was injected simultaneously at two sites in each everted cheek pouch of anaesthetized animals.

Tumours were periodically calibrated using the formula $V = 4/3\Pi a^2 b/_2$, where *a* is the shortest dimension and *b* is the longest dimension (Hibasami & Ito, 1981).

The cost of the equipment described here for the production of cutaneous vesicles is less than 10% that of the commercially available Dermovac (Instrumentarium, Helsinki, approx. $785). In addition, the apparatus can be used on multiple animals simultaneously and in a fraction of the recommended time (15 min *vs.* 2 h).

An inoculum of only $2.5 \times 10^5$ cells produced tumour nodules 100% of the time in the cheek pouch and in the skin within 24 h. Although growth kinetics were similar in both groups during the first 12 days, at Day 16 the intracutaneous tumours were 20% smaller than the cheek pouch ones (Figure 2).

Necrosis was first seen at 16 days in 4/19 cheek pouch tumours; by 21 days all cheek pouch tumours had some evidence of necrosis. In this group no correlation was found between the onset of necrosis and size. No ulceration and no visible necrosis on sectioning were observed in the intracutaneous tumours to 40 days after inoculation.

**Figure 2** Volume of growing tumours in the intracutaneous nodules (○) and in the cheek pouch (●), days after inoculation. Data points are given ±s.e.

Although intracutaneous tumours were consistently spherical (Figure 3), there was no uniformity of shape of the cheek pouch tumours. The disparity in shapes of the cheek pouch tumours became more prominent 48 h after inoculation. In

**Figure 3** Transplanted tumours. Four transplanted pancreatic tumours (arrows) growing on hamster back 8 days after inoculation.

the intracutaneous model, 4/20 tumours (all 4 in the same animal) involuted between 12–16 days. Only one cheek pouch tumour disappeared; the rest of the nodules continued to grow. Involuted tumours were excluded from the final analysis.

There are several advantages of the intracutaneous model: clear visualization and easy accessibility of the tumour nodules, reproducible sizes of identical shape, no necrosis at 40 days for fast growing tumours, and no anaesthesia requirement for the accurate measurement of tumour size. The disadvantage of this method is that it is not immunologically privileged as the hamster cheek pouch or the anterior chamber of the eye (Billingham & Silvera, 1971; Gimbrone *et al.*, 1974).

Intracutaneous inoculation of tumours seems to be a simple, inexpensive, efficient and reproducible system for growth of transplantable cells.

**References**

BILLINGHAM, R. & SILVERA, W. (1971). Immunologically privileged sites. In *The Immunology of Transplantation*, p. 69. Prentice-Hall: New Jersey.

GIMBRONE, M.A., COTRAN, R.S., LEAPMAN, S.B. & FOLKMAN, J. (1974). Tumor growth and neovascularization: An experimental model using rabbit cornea. *J. Natl Cancer Inst.*, **52**, 413.

HIBASAMI, H. & ITO, H. (1981). Antitumor effect of dicyclohexylammonium sulfate, a potent inhibitor of spermidine synthase. *Gann*, **72**, 512.

KÜSTALA, N. (1968). Suction blister device for separation of viable epidermis from dermis. *J. Invest. Dermatol.*, **50**, 129.

LEVINE, N., QUEEN, L., CHALOM, A.A. & DANIELS, L.J. (1982). Animal model for intracutaneous melanoma. *J. Invest. Dermatol.*, **78**, 191.

MIDDLETON, M.C. (1980). Evaluation of cellular injury in skin using enzyme activities in suction blister fluid. *J. Invest. Dermatol.*, **74**, 219.

TOWNSEND, C.M., JR., FRANKLIN, R.B., GELDEN, F.B., GLASS, E. & THOMPSON, J.C. (1982). Development of a transplantable model of pancreatic duct adenocarcinoma. *Surgery*, **92**, 72.